# Insufficient evidence for BMAA transfer in the pelagic and benthic food webs in the Baltic Sea

**DOI:** 10.1038/s41598-019-46815-3

**Published:** 2019-07-18

**Authors:** Nadezda Zguna, Agnes M. L. Karlson, Leopold L. Ilag, Andrius Garbaras, Elena Gorokhova

**Affiliations:** 1 Department of Environmental Science and Analytical Chemistry, Stockholm University, Stockholm, Sweden; 2 Department of Ecology, Environment and Plant Science, Stockholm University, Stockholm, Sweden; 3 Mass Spectrometry Laboratory, Center for Physical Science and Technology, Vilnius, Lithuania

**Keywords:** Chemical ecology, Natural hazards

## Abstract

The evidence regarding BMAA occurrence in the Baltic Sea is contradictory, with benthic sources appearing to be more important than pelagic ones. The latter is counterintuitive considering that the identified sources of this compound in the food webs are pelagic primary producers, such as diatoms, dinoflagellates, and cyanobacteria. To elucidate BMAA distribution, we analyzed BMAA in the pelagic and benthic food webs in the Northern Baltic Proper. As potential sources, phytoplankton communities were used. Pelagic food chain was represented by zooplankton, mysids and zooplanktivorous fish, whereas benthic invertebrates and benthivorous fish comprised the benthic chain. The trophic structure of the system was confirmed by stable isotope analysis. Contrary to the reported ubiquitous occurrence of BMAA in the Baltic food webs, only phytoplankton, zooplankton and mysids tested positive, whereas no measurable levels of this compound occurred in the benthic invertebrates and any of the tested fish species. These findings do not support the widely assumed occurrence and transfer of BMAA to the top consumers in the Baltic food webs. More controlled experiments and field observations are needed to understand the transfer and possible transformation of BMAA in the food web under various environmental settings.

## Introduction

The neurotoxin β-N-methylamino-L-alanine (BMAA), a naturally produced non-proteinaceous amino acid, has been implicated in the etiology of amyotrophic lateral sclerosis–parkinsonism-dementia complex (ALS–PDC) for the last 60 years^1^. A causative association between dietary exposure to BMAA and this pathological condition has been broadly discussed^2^, which stimulated research on BMAA production and biomagnification in food webs and development of analytical approaches for detection and quantification of BMAA and its natural isomers, 2,4-diamino butyric acid (DAB), β-amino-N-methyl-alanine (BAMA) and N-(2-aminoethyl) glycine (AEG)^3,4^.

Great analytical efforts have been taken to improve and standardize sample preparation and analytical procedures, including using internal standards for accurate identification and quantification of BMAA and its isomers in biological samples^5^. The evaluation of the analytical techniques strongly suggest that some of the previous data on BMAA occurrence overestimate the BMAA concentrations in the environmental samples and, perhaps, misidentify BMAA producers^6^. At first, BMAA production was linked to aquatic and terrestrial cyanobacteria, both free-living and symbiotic, which were considered the only producers of this compound^3,7^. These early studies claiming that most cyanobacteria produce BMAA used liquid chromatography with fluorescence detection after derivatization of BMAA with the fluorescent derivatizing agent AccQ-Tag^7^. BMAA has been detected in a variety of aquatic environments where cyanobacteria blooms can occur, such as oceans, lakes and desert springs, but also in terrestrial environments, like desert mats^7–12^. Many of these reports, however, were disconfirmed when analytical methods for the detection and quantification of BMAA have been improved and it has been demonstrated that BMAA is not as common in cyanobacteria as previously thought^4^. Thus, much of the early research on BMAA detection used inappropriate methodology and was lacking solid evidence to support the widely reported occurrence and high concentrations of BMAA^3^. This has also been acknowledged in the toxicological^2,13^ and bioaccumulation^14^ aspects of BMAA research.

Recent studies using direct detection of BMAA with liquid chromatography and tandem mass spectrometry have shown that most cyanobacteria do not produce BMAA or produce very low quantities^15^. Moreover, some cyanobacteria were found to contain DAB, a different amino acid neurotoxin, earlier associated with grass pea^16^. Owing to the differences between the analytical methods^6^, the reported concentrations even within the cyanobacteria, the focal group of BMAA producers, vary by orders of magnitude among the studies^3^. Currently, the method of choice employs AccQ-Tag Reagent Kit (AQC) derivatization and LC-MS/MS detection in selected reaction monitoring (SRM) mode^17^. This configuration has been shown very sensitive and most suitable for analyses of complex samples^18^.

The current view is that BMAA supposedly originates from some microalgae and cyanobacteria. In addition to the latter, several species of diatoms and dinoflagellates were found to also produce BMAA^19–23^. As diatoms and dinoflagellates are the major contributors to primary production in oceans, with their spring and fall blooms being a regular feature in most temperate systems^24^, the list of potential BMAA sources in aquatic food webs has been greatly expanded as well as the production capacity. These findings have also increased the possible variety of BMAA routes and bioaccumulation pathways in ecosystems^3^.

In various freshwater and marine environments, invertebrate grazers and fish exposed to blooms of potential BMAA producers have also been analyzed and, at least in some studies, found to accumulate BMAA in relation to lower trophic levels^22,23,25,26^. However, negative outcomes of such surveys are also quite common^27,28^. Then again, most studies reporting BMAA transfer from primary producers to fish have weak sampling design, suffering inconsistencies in both temporal and spatial correspondence between the collected samples of sources and consumers^10,29^. These inconsistencies hamper quantitative analysis of the BMAA transfer in the food web and make it difficult to trace this compound to specific producers. As a result, controversy surrounds the sources and pathways of BMAA production and accumulation in aquatic ecosystems, which is further complicated by the inadequate analytical approaches that have been frequently used in the past^3^.

The seasonal cyanobacterial blooms^30^ and detection of BMAA in the Baltic Sea biota raised interest in the BMAA production and fate in this system^18,19,29^. Current reports of BMAA occurrence in the Baltic region suggest that benthic fish have higher BMAA levels than pelagic ones^31^ even though no BMAA was found in sediments^19^. Unfortunately, most of the evidence for the trophic transfer and bioaccumulation of BMAA is based on the samples of seston/sediment and consumers collected outside the spring (diatoms and dinoflagellates) and summer (cyanobacteria) bloom periods, and in the areas not subjected to intense cyanobacterial blooms, such as the west coast of Sweden^19,32^.

One would expect that pelagic fish that feed on zooplankton grazing on diatoms, dinoflagellates, and cyanobacteria (i.e., known BMAA producers) in the water column would have higher BMAA concentrations in their body tissues compared to the bottom-dwelling fish that feed primarily on deposit-feeding benthic animals which consume at least partially degraded material. Indeed, even though spring diatom bloom is settled as a relatively non-degraded material^33^, the microbial activity in the sediment would decrease BMAA content of the algae considering that a suite of models based on chemical structure suggests that this compound is readily biodegradable^34^. Therefore, generally lower BMAA levels in the benthic food webs can be expected. The settling of the summer bloom of cyanobacteria to the sediment has earlier been considered negligible^35^, although more recent studies show that other cyanotoxins can accumulate in sediments and become transferred to benthic food chains^36^. To clarify the relative importance of pelagic and benthic pathways of BMAA, data from a relatively narrow geographic location for the relevant sources and consumers (i.e., comprising a common food chain) and at the proper time period (i.e., before and after bloom or within an ecologically meaningful time period) are needed.

Unambiguous evaluations of BMAA transfer and biomagnification in nature are virtually non-existent because BMAA concentrations in prey are often compared with those in potential consumers without knowledge of feeding relationships and trophic status. The study of trophic transfer requires discriminating food webs and ascribing trophic position to organisms. Stable isotope ratios of carbon (^13^C: ^12^C; δ^13^C) and nitrogen (^15^N: ^14^N; δ^15^N) are now recognized as a standard method in ecology and ecotoxicology surveys aiming to elucidate bioaccumulation pathways^37,38^. As a result of differential fractionation of light and heavy isotopes during food assimilation and metabolism, δ^13^C can be used to identify food sources (if they have distinct isotopic signatures), whereas δ^15^N can be used for inferring the relative trophic position of a consumer within a food web. Comparing data on naturally produced toxin^39^ or pollutant^38^ concentrations with trophic levels inferred from stable isotope methodologies can enhance our understanding of trophic and contaminant relationships in aquatic biota.

**Figure 1:**
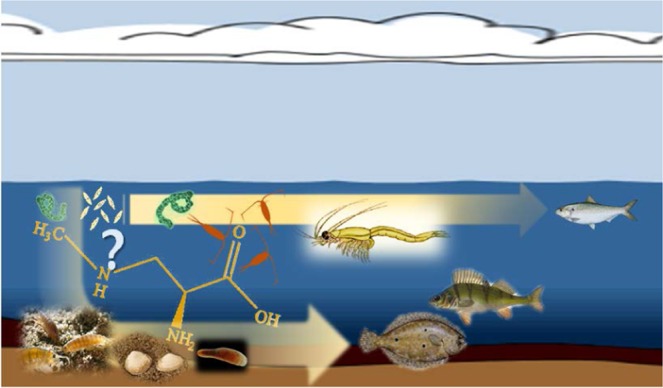
Conceptual diagram of the study design and the potential role of pelagic (invertebrates: zooplankton and mysids, and fish: herring) and benthic (invertebrates: amphipods, clams, priapulids, and polychaetes, and fish: flounder and perch) food chains in the BMAA transfer from primary producers.

To elucidate BMAA distribution in the Baltic ecological pathways (Fig. 1) in relation to phytoplankton development, we analyzed BMAA levels in the pelagic and benthic food webs using state-of-the-art analytical approach for BMAA detection and quantification. In parallel, to confirm the trophic positions of the food web components used for the BMAA analysis, their stable isotope (δ^13^C and δ^15^N) composition was determined.

## Material and Methods

### Test system and food webs

The Baltic Sea is a brackish estuary, with gradually decreasing salinity from its entrance in the southwest to the inner parts. As a result of this salinity gradient, marine species that reproduce in freshwater tributaries live side by side with freshwater species that can tolerate the brackish conditions. In this area, three bloom events occur annually, in spring, summer and autumn. The early spring bloom consist almost exclusively of diatoms (mainly *Skeletonema costatum* and *Thalassiosira baltica*), whereas the late spring bloom is largely composed by dinoflagellates (*Scrippsiella*/*Biecheleria*/*Gymnodinium* complex, *Peridiniella catenata*, and *Heterocapsa triquetra* as regular dominant species)^40^. The summer blooms of diazotrophic filamentous cyanobacteria are strongly dominated by *Aphanizomenon* sp., *Nodularia spumigena* and *Dolichospermum* spp.^41^.

**Figure 2:**
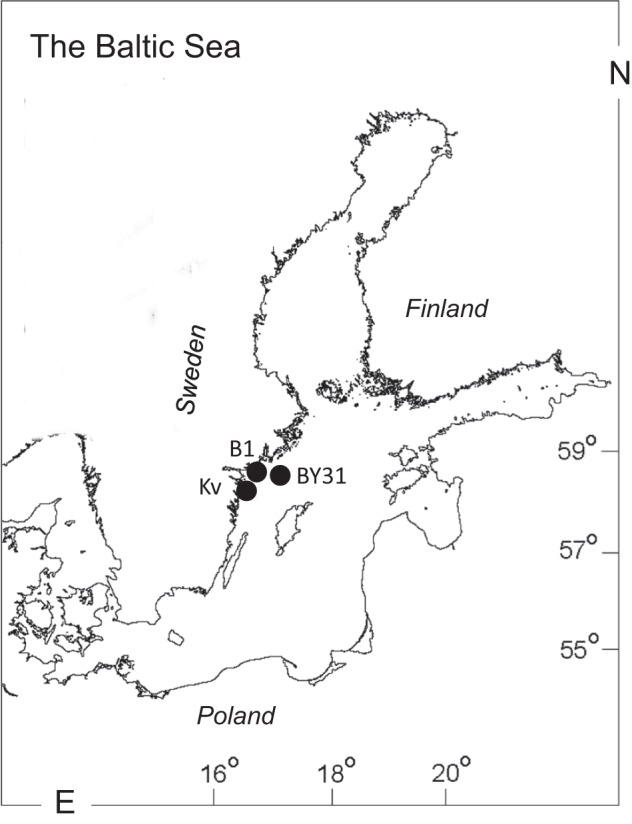
Sampling locations in the norther Baltic Proper for food chain components representing pelagia (BY31, Landsort Deep) and benthic system (B1 and Kvädöfjärden, Kv, both in the viscinity of the Askö Field Station). In this study area, the salinity is around 6 practical salinity units (PSU). See Fig. S1 (Supplementary Information) for the detailed map of the Askö area with stations used for collecting benthic invertebrates and sediment.

As potential BMAA sources, early- and late-summer phytoplankton communities were used. To represent the pelagic chain of consumers, we used mesozooplankton (copepods and cladocerans) and zooplanktivores, mysids (omnivores, feeding on phyto- and zooplankton) and herring (feeding mainly on zooplankton). The benthic chain consumers were invertebrates (amphipods, priapulids, polychaetes, and clams) and nekto-benthivorous fish (perch and flounder) (Table 1; Fig. 1). The same sources and consumers were used for δ^13^C and δ^15^N composition. All sampled food web components were selected to capture relatively narrow geographic (Fig. 2) and temporal (June-September 2010) variability.

**Table 1:** Summary of samples used for BMAA analysis and SIA; *n* refers to the number of replicates.

Source	Location	Date	Collection method and sample preparation	Organism size	BMAA (*n*)	SIA(*n*)
**Benthic food chain**						
Sediment	Askö area (see Fig. S1)	7 Jun 28 Jun 23 Sep	Upper 1–2 cm of sediment were collected with a benthic sled; the macrofauna were extracted with 1-mm sieve and used as benthic samples. The sediment was frozen in bulk at −20 °C	Not applicable	6	5
Amphipod *Monoporeia affinis*			Sieved from the sediment, sorted by species and size class based on the visual inspection and frozen in bulk at −20 °C	4.2 mg DW/ind.	6	3
Baltic clam *Macoma baltica*				9.3 mg DW/ind.	6	5
Priapulid *Halicryptus* spp.				15.8 mg DW/ind.	4	6
Polychaete *Marenzelleria* spp.				11.1 mg DW/ind.	5	4
Flounder *Platichthys flesus*	Kvädö-fjärden	12 Sep	Collected within SNMMP and stored at −30 °C in the Environmental Specimen Bank of the Swedish Natural History Museum	21.3 cm	3	5
Perch *Perca fluviatilis*				19.2 cm	3	3
**Pelagic food chain**						
Phytoplankton	Landsort Deep	19 Jun 8 Aug	Tows were taken with a small plankton net (10 µm) in the upper 20 m. Crustacean zooplankton (*Acartia* spp., *Eurytemora affinis*, and *Bosmina coregoni*) were separated by a light trap, picked with forceps under a compound microscope, and frozen at −20 °C. The rest of the material was filtered (5 µm) and used as phytoplankton samples (>20 mg wet mass sample^−1^). As detritus and heterotrophic protists were present, these samples were also referred to as *seston*.	>10 µm	5	2
Zooplankton		19 Jun 8 Aug		18.1 µg DW/ind.	5	4
Mysid *Mysis mixta*		12 Jun 15 Aug	Sampled with a WP2 (100 µm) plankton net in the upper 100 m, identified to species level and frozen individually in Eppendorf tubes at −20 °C.	5.5 mg DW/ind.	4	4
Mysid *Neomysis integer*				3.9 mg DW/ind.	4	3
Herring *Clupea harengus*		16 Sep	Collected within SNMMP and stored at −30 °C in the Environmental Specimen Bank of the Swedish Natural History Museum.	16.7 cm	3	5

Organism size for the benthic invertebrates and mysids was determined as the average dry mass per individual in the SIA samples and the number of individuals used to prepare these samples; for the fish, it is the total body length. For BMAA; at least two technical replicates were used.

Sediment and invertebrates comprising the benthic food chain were collected in a coastal area, and those for the pelagic food chain were collected in the open sea area of the Baltic proper (Fig. 2). The benthic chain samples were collected at several sites in the vicinity of the Askö Field Station (station B1, 58°48′28 N, 17°37′60 E; Swedish National Marine Monitoring Program, SNMMP) in the north-western Baltic proper. Sediment and macrofauna were collected at stn Håldämman (30 m depth, 58°49′18 N, 17°34′58 E) and stn Uttervik (20 m, 58°50′58 N, 17°32′77 E) on 2–3 sampling events in June and September (Fig. S1, Supplementary Information). We used a benthic sled, set to collect the top 1–2 cm sediment, which was then sieved through a 1-mm sieve to retain macrofauna. The species selected were the bivalve *Limecola balthica*, the non-indigenous polychaete *Marenzelleria arctia*, the amphipod *Monoporeia affinis*, and the priapulid worm *Halicryptus spinulosus*. The sieved sediment samples and the recovered animals were frozen and stored at −20 °C until analyses (Table 1).

**Figure 3:**
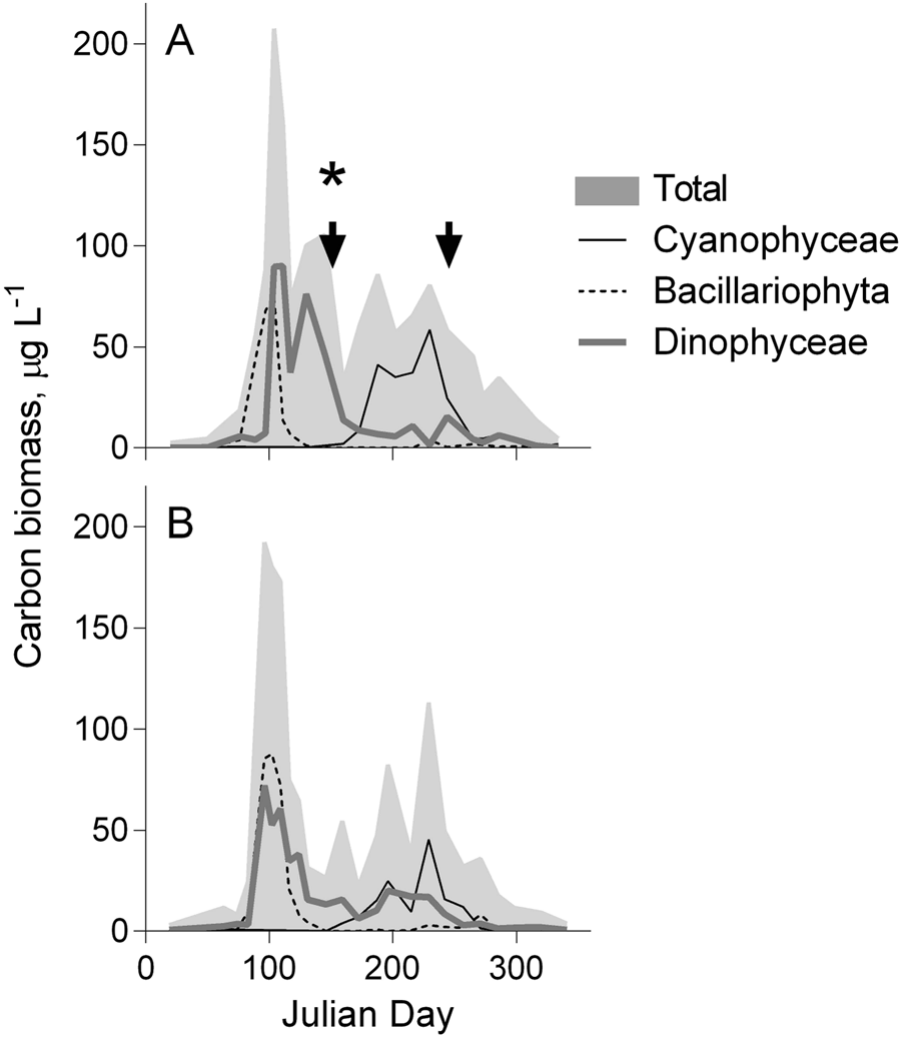
Phytoplankton dynamics in the study areas: (**A**) the Landsort Deep (pelagic food chain) and (**B**) Askö station (benthic food chain). The total carbon biomass (μg L^−1^) of phytoplankton (>3 μm); and seasonal dynamics of the main BMAA producers (cyanobacteria, diatoms and dinoflagellates) in the year 2010 are obtained from the SHARK database. Sampling occasions are indicated by arrows and the BMAA-positive sample with asterisk.

**Figure 4:**
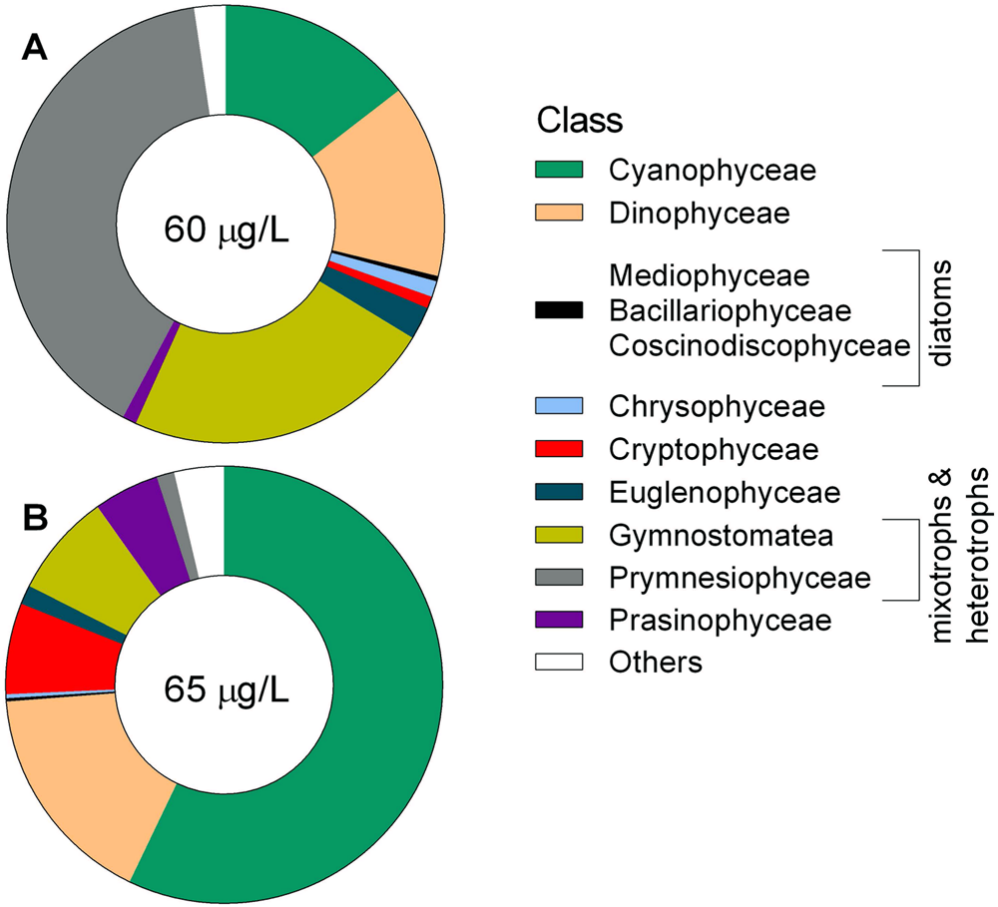
Composition of the phytoplankton communities that were (**A**) BMAA-positive (June) and (**B**) BMAA-negative (August) presented as relative contribution of main phytoplankton classes by carbon biomass (μg L^−1^). Number in the middle of the pie diagram is the total carbon biomass of the phytoplankton at the time of collection.

The pelagic samples (phytoplankton, zooplankton, and mysids) were collected in the Landsort Deep (open-sea stn BY31; 459 m, 58°35′00 N, 18°14′00 E; SNMMP) on 3–4 sampling occasions in June and August-September 2010. Phytoplankton samples were taken using a plastic hose lowered to 20 m depth, giving an integrated 0–20 m sample. After mixing in a bucket, the phytoplankton was filtered on a GF/F (0.7-µm) to gather sufficient material for the analysis. The filters were folded, frozen and stored at −20 °C until analyses. Complementary data on phytoplankton (cell size ≥3 µm; Figs 3 and 4) taxonomic composition and carbon biomass were obtained from the SHARK database at the Swedish Meteorological and Hydrological Institute (SMHI; www.smhi.se). Here, the taxonomic data are presented with particular focus on cyanobacteria (class Cyanophyceae), dinoflagellates (class Dinophyceae) and diatoms (classes Mediophyceae, Bacillariophyceae, and Coscinodiscophyceae); other phytoplankton were also grouped at the class-level. The samples used for the analysis of phytoplankton community structure were collected within 3–4 days of our sampling occasions, and the community composition was assumed to represent that in our samples analyzed for BMAA. Zooplankton were collected by vertical tows in the upper 30 m using a 90-µm WP-2 net (diameter 57 cm). Species that dominated, the copepods (*Acartia* spp. and *Eurytemora affinis*) and the cladocerans (*Bosmina coregoni maritima*), were picked under a stereomicroscope to avoid contamination with phytoplankton and frozen in bulk. The rest of the single-tow sample was preserved in 4% borax buffered formaldehyde for species identification and community analysis. Mysids (*Mysis mixta* and *N. integer*) were collected in the upper 100 m using either a 500- or a 200-µm WP-2 net (diameter 57 cm), length-measured, and frozen individually in Eppendorf tubes (Table 1).

Fish were obtained from the Environmental Specimen Bank of the Swedish Museum of Natural History (http://www.nrm.se/en/forskningochsamlingar/miljoforskningochovervakning/miljoprovbanken.9000848.html). The collection was made in September 2010 as a part of the Swedish National Monitoring Program for Contaminants in Marine Biota. Bottom-feeding fish species included the European flounder *Platichthys flesus*^42^ and the Eurasian perch *Perca fluviatilis*^43^; both species were sampled in Kvädöfjärden (58.0497° N, 16.7831° E). As a fish with pelagic feeding, we used the Baltic herring *Clupea harengus*^44^ sampled in the Landsort Deep. All fish was length-measured, the epidermis and subcutaneous fatty tissue were carefully removed, and muscle tissues were taken from the middle dorsal muscle layer for BMAA and stable isotope measurements (Table 1).

### Positive controls

Two positive controls were used: (1) a blue mussel (*Mytilus edulis*; Bivalvia) sample that tested positively in the previous study^18^ and (2) cyanobacterium *Nodularia spumigena* (strain UHCC 0039, formerly named *N. spumigena* AV1, a Baltic Sea isolate)^45^ grown axenically in culture and harvested at exponential and stationary growth phases. The cyanobacterium was grown in a modified Z8 nutrient solution in 16:8 h light:dark regime (40 µE m^−2^s^−1^) at 20–21 °C with constant shaking and harvested when cell density reached approximately 5 × 10^6^ cell/mL (3–5 days) and 10^9^ cell/mL (9–10 days) for the exponential and stationary phase samples, respectively; three replicates for each group were obtained. The material was harvested by filtration and processed in the same way as the field-collected phytoplankton samples.

### Analysis of total BMAA

Samples for the UPCL-MS/MS analysis of total BMAA were prepared according to Jiang and co-workers^5,18^, with some modifications. The sample material (seston, shell-free tissues of clams, whole bodies of invertebrates and dissected fish muscle tissue) was homogenized with a mortar and pestle with the addition of liquid nitrogen. A weighed subsample of the homogenate (20 to 50 mg; Mettler Toledo XP6; +/− 0.001 mg) was mixed with 600 µL of water and subjected to ultrasound treatment with Vibra CellTM, Sonics & Materials Inc. Danbury CT, USA (3 min, 1 second on/off pause, 70% amplification) in an ice-water bath. For each sample, a standard amount of material was analyzed by using an aliquot corresponding to 10-mg of the homogenate that was transferred to a glass hydrolysis vial together with 10 µL of deuterated BMAA standard, water, and 6 M HCl, and hydrolyzed at 110 °C for 20 h. Thus, the amount of the analyzed material was kept constant (10 mg wet mass sample^−1^) for all samples and controls.

The hydrolyzed samples were filtered in a centrifuge using spin filters (0.2 µm, Thermo Scientific, USA), dried under nitrogen flow, reconstituted in 1 mL of MilliQ water, and subjected to a two-step clean-up: (1) liquid-liquid extraction with chloroform to remove lipids and other hydrophobic compounds, and (2) solid phase extraction (SPE) with Isolute HCX-3 column (Biotage Sweden AB) using 1 mL of 0.1% formic acid in water and 1 mL of methanol for column conditioning followed by sample application and washing by 1 mL of methanol and 1 mL of 0.1% formic acid in water. For elution (two rounds), 0.8 mL of 2% NH_4_OH in methanol were used. After the SPE step, the samples were dried under nitrogen flow.

Samples were derivatized with 6-aminoquinolyl-N-hydroxysuccinimidyl carbamate (AQC) reagent using AccQ-Tag kit (Waters; WAT052880, Milford, USA). Dried samples were reconstituted in 20 µL 20 mM HCl, and mixed with 60 µL borate buffer (from AccQ-Tag kit) and 60 µL of AQC reagent. Before analysis, all samples were dried and reconstituted in the initial UHPLC mobile phase (30 µL of 5% acetonitrile in water). All samples were analyzed in duplicates.

The samples were analyzed by UHPLC-MS/MS as described elsewhere (Jiang *et al*. 2012) using TSQ Vantage triple quadrupole mass spectrometer (Thermo Fisher Scientific, USA) equipped with an Accela pump and auto-sampler as well as a degasser. An additional pump (Rheos 4000 pump, Flux instruments) was used for post-column addition of 0.3% acetic acid in acetonitrile at the flow rate of 600 µL/min. The UHPLC system was equipped with an ACCQ-TAG^TM^ ULTRA C18 column (100 × 3 × 2.1 mm, 1.7 mm particle size, Waters, Ireland). Binary mobile phase (MP) was delivered according to the programmed gradient (MP A: 0.3% acetic acid and 5% acetonitrile in water and MP B: 0.3% acetic acid in acetonitrile) as follows: 0.0 min, 0% B (flow rate 200 µL/min); 10.0 min, 10% B (flow rate 400 µL/min for the rest of the program); 11.0 min, 80% B; 12.0 min, 80% B; 12.1 min, 0% B; and 16.0 min, 0% B. MS/MS analysis was performed by multiple reaction monitoring (MRM) in positive ion mode. One monitored MRM transition (459.18 > 119.08) was general for BMAA and its isomers (BAMA, DAB and AEG), whereas other three MRM transitions were diagnostic for the particular isomers (459.18 > 258.09 for BMAA and BAMA, 459.18 > 188.08 for DAB, and 459.18 > 214.10 for AEG), and one MRM transition was unique for d3-BMAA internal standard (462.20 > 122.10). The unique transitions in combination with carefully compared retention times and peak area ratio between the general and diagnostic product ions (ratio 119/258 equal to 4.4 ± 10%) allowed a reliable detection of BMAA. The BMAA quantification was done based on peak area of *m*/*z* = 119.

A calibration curve for the range of concentrations (0.1–10 ng BMAA on column) showed excellent linearity (R^2^ > 0.99) and was used for BMAA quantification. Method-specific limit of detection (LOD) and limit of quantification (LOQ) were previously estimated to be below 0.01 µg BMAA g^−1^ wet mass^18^ by analyzing spiked samples of crayfish muscle tissue. The accuracy and precision (108–119% and <15% RSD, respectively) were evaluated in the same study^18^ with the quality control samples at different BMAA concentrations. The sensitivity of the method was determined as low as 4.2 fmol BMAA/injection^18^.

In the current study, the method performance was confirmed using spiked matrix of cod muscle, which was proven to be negative for BMAA in the previous studies^6,18^. The spiked sample was analyzed in triplicate, and the recovery was 106%, which is in agreement with the previously published method performance report^18^. The lowest concentration spiked was 0.05 ng/10 mg wet mass, and this amount was detected S/N > 10 for *m*/*z* = 119.

### SIA analysis

All biological material and sediment were dried to a constant weight (24 to 60 h, depending on the sample type) at 60 °C. To prepare the samples of zooplankton, phytoplankton, and sediment, the bulk material was used, whereas, for the samples of fish and invertebrates, we subsampled ~0.7 mg dry weight (DW). The samples were transferred to tin capsules, dried again at 60 °C for 24 h, and shipped to the Center for Physical Science and Technology, Vilnius, Lithuania. The SIA was conducted using Flash EA 1112 Series Elemental Analyzer that was connected via a Conflo III to a DeltaV Advantage Isotope Ratio Mass Spectrometer (all Thermo Finnigan, Bremen, Germany). Ratios of ^14^N: ^15^N and ^12^C: ^13^C were expressed relative to the international standards, atmospheric air (N) and Pee Dee Belemnite (C) and presented in a δ-notation, parts per thousand difference from the standard. Internal reference (cod muscle tissue prepared in the same way as the test samples) was analyzed every 20 samples; the analytical precision was 0.1‰ for both δ^15^N and δ^13^C.

### Ethics statement

All fish samples were collected by the Swedish Museum of Natural History in accordance with the national legal requirements and approved by the Swedish Board of Agriculture (Stockholms norra djurförsöksetiska nämnd; permit number 83/14).

## Results and Discussion

No measurable levels of BMAA structural isomers (AEG, DAB and BAMA) were detected in either controls or the samples (Fig. S2, Supplementary Information). With regard to BMAA, the positive controls performed adequately. The blue mussel positive control yielded 0.088 ± 0.008 µg g^−1^ wet mass (mean ± SD, n = 2), which is within the range of the BMAA concentration observed previously in the blue mussels from the same batch (0.08–0.90 µg g^−1^ wet mass)^18^. In the *Nodularia spumigena* harvested at the exponential and stationary phase, the BMAA concentration (mean ± SD, n = 3) was 0.98 ± 0.06 and 0.13 ± 0.02 µg g^−1^ wet mass, respectively. These concentrations are comparable to those reported for other cyanobacteria^5,46^. Thus, both types of the control material were found to contain the expected amounts of BMAA. Moreover, in the cyanobacterium, the BMAA levels were approximately 8-fold higher during the exponential growth compared to the aging culture suggesting a possibility of intrapopulation variability of BMAA levels as a function of bloom phase.

The BMAA concentrations measured in seston and zooplankton varied from 0.83 to 1.13 µg g^−1^ wet mass, which corresponds to approximately 7.5–9.5 µg g^−1^ dry mass using common wet-to-dry mass conversion factors for phyto- and zooplankton^47,48^. These values are within the range reported for algae and invertebrates in other systems by studies that used reliable quantification methods^4^. In the Baltic Sea, the BMAA concentration in cyanobacteria-rich phytoplankton community has been reported to vary from 0.001 to 0.015 μg BMAA/g dry mass, and that of zooplankton 0.004 to 0.087 μg BMAA/g dry mass^29^. These values are more than 100-fold lower than the concentrations measured in our study, but also several orders of magnitude lower than plankton BMAA concentrations reported from other – mostly freshwater – systems. For instance, samples of cyanobacteria in Dutch urban waters^46^, British^49^ and South African^50^ lakes as well as Taihu Lake in China^25^ are in the µg BMAA/g dry mass range or higher, which is two to five orders of magnitude higher than the range reported by Jonasson and co-workers^29^. Other studies^51^ also point out 50- to 1000-fold higher BMAA concentrations in invertebrates compared to those reported by Jonasson *et al*.^29^. A reason for these conflicting results can be related to the fact that BMAA quantification in the latter study was based on a semi-quantitative method, with no internal standard; moreover, no LOD values were reported. The method that was employed involves a clean-up procedure based on the liquid-liquid extraction followed by a solid phase extraction, which can introduce a significant loss of BMAA during the work-up. As shown by the inter-laboratory evaluation^52^, the recovery of this clean-up procedure might be as low as <10%. The estimated loss of analyte during the sample preparation in the current study as well as regular checks was around 60%, i.e., similar to that reported by Jiang and co-authors^5^, who developed the method. Therefore, the addition of an internal standard before the clean-up is required to control for the losses during the sample work-up.

**Figure 5:**
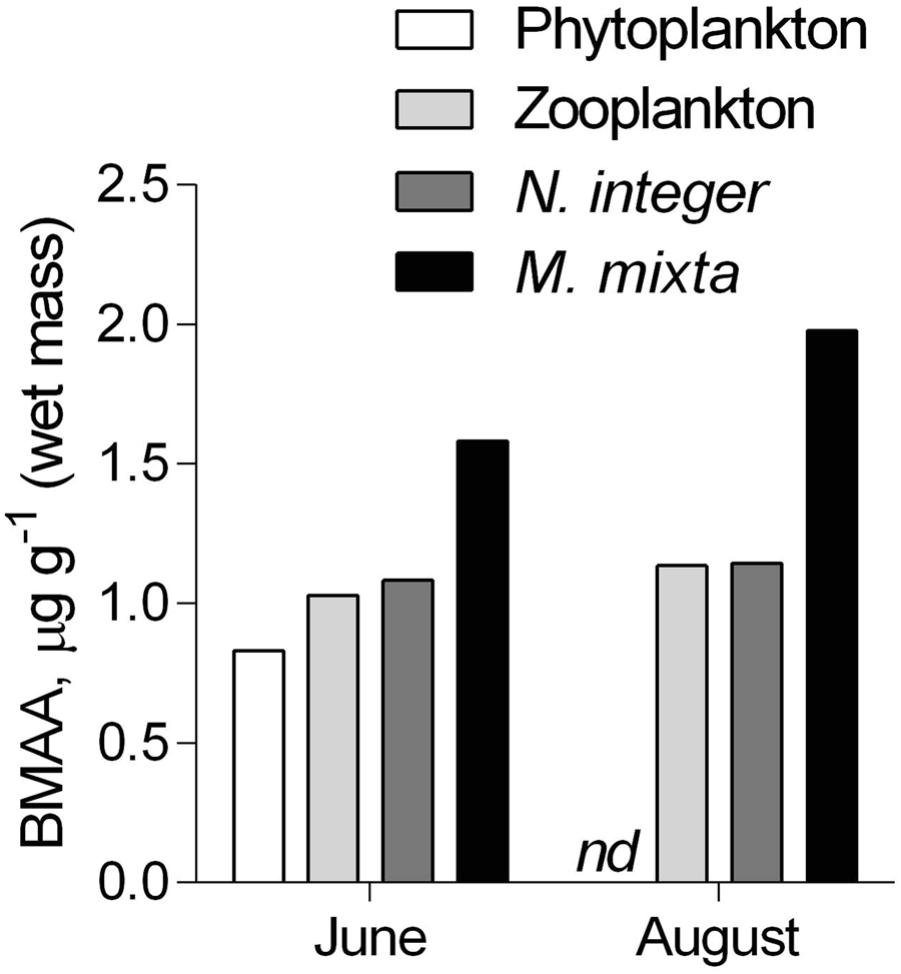
BMAA concentration (μg g wet weight^−1^) in phytoplankton, zooplankton, and mysids (*Neomysis integer* and *Mysis mixta*) collected before (June) and after (August) the cyanobacterial bloom. All samples were composite to integrate respective food web components over several sampling occasions; nd – not detectable.

Only a single phytoplankton sample was found to contain BMAA, and the concentration measured was within the levels reported in studies that are considered as truly reliable with regard to the quality assurance and the protocols involved^4^. The phytoplankton samples were taken during two periods: (1) past the spring bloom comprised by diatoms and then dinoflagellates but before the onset of the summer cyanobacteria bloom (June), and (2) after the cyanobacteria bloom collapse (August; Fig. 3A). Both spring and summer blooms were of average magnitude and duration in relation to the multiyear variability^24,41^ and similar between the coastal and open sea areas (Fig. 3). However, only one phytoplankton sample taken in the mid-June contained BMAA (Figs 4A, 5). The phytoplankton community composition at the time of sampling was typical for this area and the time of year, with biomass composed of ~40% haptophytes, 23% ciliates, 16% cyanobacteria, 15% dinoflagellates, the rest being a mixture of summer species (Fig. 4A). Thus, the community had a high contribution of ciliates and haptophytes (64% together), i.e., mixotrophic and heterotrophic protists with a higher trophic position than the autotrophic phytoplankton. The August samples that did not yield measurable BMAA quantities had approximately 3.5-fold higher percentage of cyanobacteria (Fig. 4B) and 8-fold lower percentage of bacterivorous species. In no case the contribution of the diatoms exceeded 0.5%. Given this variability in the taxonomic composition of the phytoplankton, with two potentially significant BMAA producers (i.e., cyanobacteria, and dinoflagellates) being moderately abundant, we cannot identify a plausible source of the BMAA in our sample. It is also possible that during the decline of the bloom, the cyanobacteria have lower BMAA levels as what we found in our positive control samples collected during the stationary phase; this might have contributed to the non-detectable levels in the August sample.

It is also possible that heterotrophic protists can accumulate BMAA and contribute to its bulk concentration in seston. It has been speculated that picocyanobacteria, such as *Synechococcus*, can contribute to BMAA production and transfer to primary consumers^53^. Under culture conditions, *Synechococcus* has been found to produce DAB but not BMAA^23^; however, virtually nothing is known about picoplankton contribution to the field-observed levels of BMAA and its isomers. In the microbial food web, the phagotrophic protists (heterotrophic ciliates and mixotrophic phytoplankton) feeding on bacterioplankton, including picocyanobacteria, may accumulate BMAA and transfer it further to the primary consumers, such as zooplankton^54^. Our findings support this hypothetical pathway, because the high contribution of phagotrophs coincided with the presence of BMAA in the seston sample, whereas neither absolute nor relative amount of filamentous cyanobacteria contributed to the BMAA levels (Fig. 4A). Analyzing more samples with different community structure and linking BMAA levels to the community composition in the microbial food web would be one way to identify the main BMAA producers and consumers in these communities. Another approach would be to use culture-based studies with species that were putatively identified as the BMAA producers in the field.

**Figure 6:**
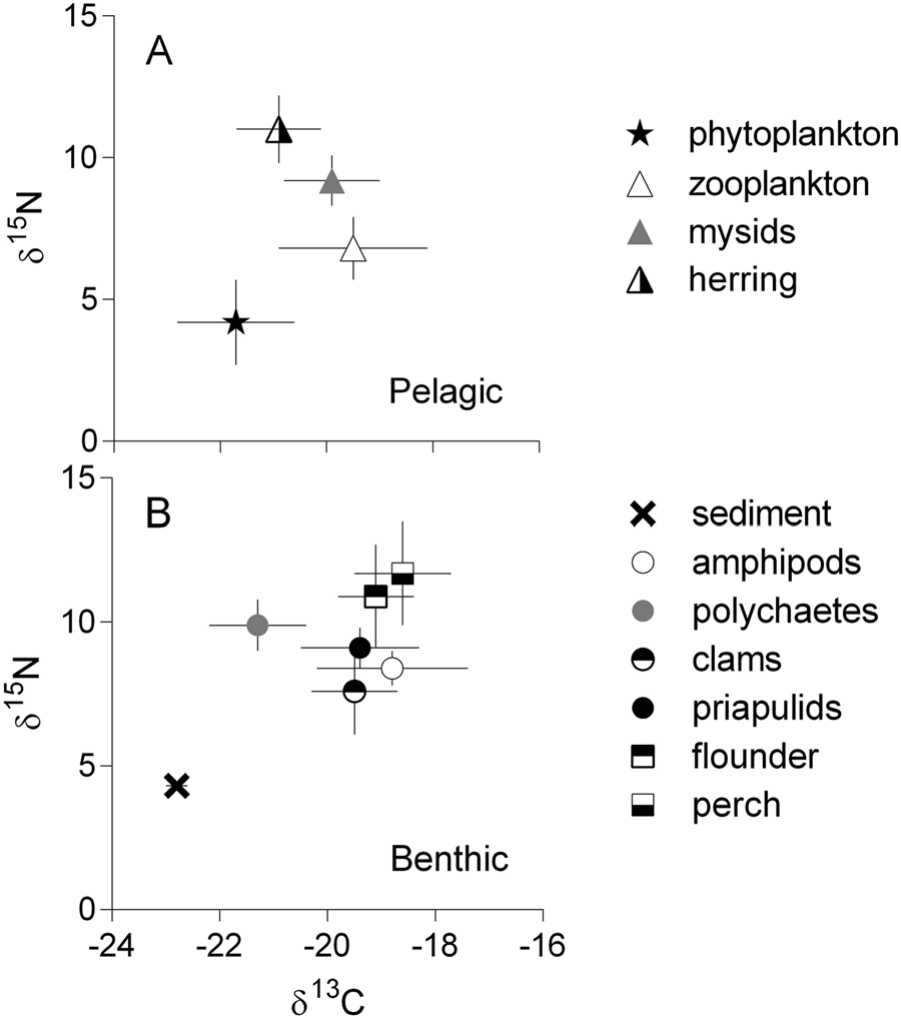
Food web structure determined by SIA (δ^15^N vs. δ^13^C; mean ± SD); pelagic (**A**) and benthic (**B**) compartments. See Table 1 for details on the food web components, sample origin, and the number of samples analyzed.

Zooplankton and mysid samples were found to contain BMAA, both before and after the cyanobacterial bloom, despite the lack of BMAA in the phytoplankton sampled in August (Fig. 5). The composition of zooplankton communities differed substantially between the sampling occasions, with cladocerans comprising >60% of the total zooplankton biomass in June and copepods (*Acartia bifilosa* and *Eurytemora affinis*) dominating (>70%) in August (data not shown). Under bloom conditions, both copepods feed selectively on ciliates^55^ which suppresses their abundance^56^. Therefore, the possibility of BMAA transfer via the microbial food web and the selective feeding on ciliates might explain BMAA presence in zooplankton in August, when no measurable levels were detected in the seston. Moreover, BMAA levels in mysids differed between the species, being 50–70% higher in *M. mixta* than in *N. integer*. The difference could be related to the more herbivorous diet of *N. integer* and more zooplanktivorous feeding of *M. mixta*^57^. These differences in diet were also supported by the δ^15^N values, which were 1.5‰ lower in *N. integer* compared to *M. mixta* (Fig. 6A), indicating more herbivorous diet of the former. Therefore, the stepwise increase in BMAA from phytoplankton to *M. mixta* (Fig. 5) could be indicative of biomagnification, albeit more data are needed to substantiate this suggestion.

It has been repeatedly stressed that when collecting material for bioaccumulation studies, it is essential to use an adequate temporal and spatial resolution and to ensure the pathway identification and trophic relatedness of the ecosystem components^58^. To confirm the trophic relationships between the organisms involved in the bioaccumulation assessment, a stable isotope approach has been commonly used^37,38^. We applied SIA to confirm the trophic linkages for the consumers, particularly the fish species with a non-sedentary behavior. The SIA data (Fig. 6) confirmed that all animals analyzed for BMAA were occupying the trophic positions as expected (based on their δ^15^N values) and belonged to the same food web (δ^13^C values). In particular, the isotopic signatures supported the assumption that herring collected in the area where zooplankton and mysids were collected was indeed relying on these prey (Fig. 2); however, we found no evidence of the BMAA presence in the fish. Previously, one out of three Baltic herring samples were tested positive for BMAA, albeit the concentration was close to LOD and LOQ^18^. As no BMAA was found in the sediment and benthic invertebrates (four species), it is not particularly surprising that both species of the benthic fish that were analyzed (flounder and perch) were negative. Previously, low BMAA concentrations were reported in the perch from the Swedish Finjasjön Lake^31^ but not the marine waters^18^, whereas no data are available on its occurrence in the flounder.

The current view that in the food webs BMAA may be transferred from producers to zooplankton and other filter-feeders, such as bivalves, and bioaccumulated in fish feeding on these invertebrates is based only on a limited data. In particular, these patterns have been reported using material collected in the Baltic Sea^29^, the Florida Bay, USA^14^, and the Portuguese transitional waters^22^. Of these studies, only the latter used a controlled sampling design, with the consumers and primary producers collected systematically from the same environment. The authors have found a correlation between the dinoflagellate *Gymnodinium catenatum* blooms and a subsequent, with a few-day lag phase, an increase of BMAA concentration in the river cockles. They have also used a laboratory experiment to demonstrate BMAA production by *G. catenatum*. In other studies, the sample material have originated from various time periods and sampling locations with no clear trophic linkage between the producers and consumers. This holds true for the samples of plankton, fish and benthic invertebrates analyzed by Jonasson and co-workers^29^, which originated from the geographically distant regions and food webs, i.e., the Baltic proper and the North Sea, which makes it questionable for the bioaccumulation assessment.

Although the bioaccumulation/biomagnification of BMAA has been broadly assumed, its mechanisms are not well understood^14^ and *in situ* evidence using material collected in a systematic way is limited. Our findings indicate that ubiquitous transfer of BMAA to the top consumers in the food webs of the Baltic Sea and, possibly, other systems, is questionable. It also implies that invertebrates and fish associated with benthic food sources in this system are less likely to accumulate BMAA compared to the pelagic food chain. However, some tissue effects on BMAA accumulation may occur resulting in higher values in, for example, protein-rich tissues/organisms. Other tissue-specific properties may also promote BMAA accumulation; for example, results from the most reliable studies show that marine bivalves are to date the matrix containing the highest amount of BMAA, far more than most fish muscles, but with an exception for shark cartilage^4^. Therefore, to interpret BMAA levels in food chains, the concentrations on the protein basis might need to be used.

To summarize, our findings suggest that BMAA levels in the Baltic food webs are low, and contrary to the reported ubiquitous occurrence of BMAA in the Baltic food webs, only phytoplankton (seston), zooplankton and mysid samples tested positive. None of the benthic invertebrates or fish species, both zooplanktivorous and benthivorous, were found to contain BMAA, which implies a low risk for the top consumers, such as seals, birds, and humans. Moreover, no measurable BMAA levels were detected in the sediment. Given that the analytical performance was adequate, and both positive controls, i.e., the blue mussel and the cyanobacterium, tested positive, we conclude that all samples that tested negative contained no measurable levels of this compound. However, our sampling was limited to a few occasions in the summer and a relatively small geographic area, whereas it is possible that BMAA levels vary depending on environmental conditions and ecological responses of its producers. Therefore, to understand the magnitude of this variability, it is important to conduct a systematic sampling throughout a year and in different parts of the Baltic Sea, collecting both biota and settling material. Sensitive and validated analytical methods should be used to ensure that the obtained results are consistent, and quality control samples must be included in the surveys to evaluate the performance of the methods, particularly the analytic recovery, LOD values, and accuracy of the results. Finally, more controlled experiments and field observations are needed to understand the toxicity and accumulation mechanisms, trophic transfer and possible transformation of BMAA in various environmental settings.

## Supplementary information

Supplementary Information

## References

[1] Cox PA, Banack SA, Murch SJ (2003). Biomagnification of cyanobacterial neurotoxins and neurodegenerative disease among the Chamorro people of Guam. Proc. Natl. Acad. Sci. USA.

[2] Chernoff N (2017). A critical review of the postulated role of the non-essential amino acid, β-N-methylamino-L-alanine, in neurodegenerative disease in humans. J. Toxicol. Environ. Health Part B.

[3] Faassen EJ (2014). Presence of the neurotoxin BMAA in aquatic ecosystems: what do we really know?. Toxins.

[4] Lance E, Arnich N, Maignien T, Biré R (2018). Occurrence of β-N-methylamino-l-alanine (BMAA) and Isomers in Aquatic Environments and Aquatic Food Sources for Humans. Toxins.

[5] Jiang L, Johnston E, Aberg KM, Nilsson U, Ilag LL (2013). Strategy for quantifying trace levels of BMAA in cyanobacteria by LC/MS/MS. Anal. Bioanal. Chem..

[6] Faassen EJ, Gillissen F, Lürling M (2012). A comparative study on three analytical methods for the determination of the neurotoxin BMAA in cyanobacteria. PloS One.

[7] Cox PA (2005). Diverse taxa of cyanobacteria produce β-N-methylamino-l-alanine, a neurotoxic amino acid. Proc. Natl. Acad. Sci. USA.

[8] Cox PA (2009). Cyanobacteria and BMAA exposure from desert dust: a possible link to sporadic ALS among Gulf War veterans. Amyotroph. Lateral Scler. Off. Publ. World Fed. Neurol. Res. Group Mot. Neuron Dis..

[9] Craighead D (2009). Presence of the neurotoxic amino acids β-N-methylamino-L-alanine (BMAA) and 2,4-diamino-butyric acid (DAB) in shallow springs from the Gobi Desert. Amyotroph. Lateral Scler..

[10] Brand LE, Pablo J, Compton A, Hammerschlag N, Mash DC (2010). Cyanobacterial blooms and the occurrence of the neurotoxin, beta-N-methylamino-l-alanine (BMAA), in South Florida aquatic food webs. Harmful Algae.

[11] Li A (2010). Detection of the neurotoxin BMAA within cyanobacteria isolated from freshwater in China. Toxicon.

[12] Metcalf JS, Banack SA, Richer R, Cox PA (2015). Neurotoxic amino acids and their isomers in desert environments. J. Arid Environ..

[13] van Onselen R, Venables L, van de Venter M, Downing TG (2018). β-N-Methylamino-L-Alanine Toxicity in PC12: Excitotoxicity vs. Misincorporation. Neurotox. Res..

[14] van Onselen, R., Downing, S., Kemp, G. & Downing, T. Investigating β-N-Methylamino-l-alanine Misincorporation in Human Cell Cultures: A Comparative Study with Known Amino Acid Analogues. *Toxins* **9** (2017). 10.3390/toxins9120400 PMC5744120 29240689

[15] Krüger T, Mönch B, Oppenhäuser S, Luckas B (2010). LC-MS/MS determination of the isomeric neurotoxins BMAA (beta-N-methylamino-L-alanine) and DAB (2,4-diaminobutyric acid) in cyanobacteria and seeds of Cycas revoluta and Lathyrus latifolius. Toxicon Off. J. Int. Soc. Toxinology.

[16] Ressler C, Redstone PA, Erenberg RH (1961). Isolation and Identification of a Neuroactive Factor from Lathyrus latifolius. Science.

[17] Spácil Z (2010). Analytical protocol for identification of BMAA and DAB in biological samples. The Analyst.

[18] Jiang L, Kiselova N, Rosén J, Ilag LL (2014). Quantification of neurotoxin BMAA (β-*N*-methylamino-L-alanine) in seafood from Swedish markets. Sci. Rep..

[19] Jiang L (2014). Diatoms: A Novel Source for the Neurotoxin BMAA in Aquatic Environments. PLOS ONE.

[20] Jiang L, Ilag L (2014). Detection of endogenous BMAA in dinoflagellate (Heterocapsa triquetra) hints at evolutionary conservation and environmental concern. PubRaw Sci..

[21] Lage S, Ström L, Godhe A, Rydberg S (2018). Kinetics of β-N-methylamino-L-alanine (BMAA) and 2, 4-diaminobutyric acid (DAB) production by diatoms: the effect of nitrogen. Eur. J. Phycol..

[22] Lage S (2014). BMAA in shellfish from two Portuguese transitional water bodies suggests the marine dinoflagellate Gymnodinium catenatum as a potential BMAA source. Aquat. Toxicol..

[23] Réveillon D (2015). β-N-methylamino-l-alanine (BMAA) and isomers: Distribution in different food web compartments of Thau lagoon, French Mediterranean Sea. Mar. Environ. Res..

[24] Klais R, Tamminen T, Kremp A, Spilling K, Olli K (2011). Decadal-Scale Changes of Dinoflagellates and Diatoms in the Anomalous Baltic Sea Spring Bloom. PLOS ONE.

[25] Jiao Y (2014). Occurrence and transfer of a cyanobacterial neurotoxin β-methylamino-l-alanine within the aquatic food webs of Gonghu Bay (Lake Taihu, China) to evaluate the potential human health risk. Sci. Total Environ..

[26] Andrýs R (2015). Improved detection of β-N-methylamino-l-alanine using N-hydroxysuccinimide ester of N-butylnicotinic acid for the localization of BMAA in blue mussels (Mytilus edulis). Anal. Bioanal. Chem..

[27] Scott PM, Niedzwiadek B, Rawn DFK, Lau BP-Y (2009). Liquid chromatographic determination of the cyanobacterial toxin beta-n-methylamino-L-alanine in algae food supplements, freshwater fish, and bottled water. J. Food Prot..

[28] Niedzwiadek B, Scott PM, Lau BP-Y (2012). Monitoring of shrimp and farmed fish sold in Canada for cyanobacterial toxins. J. Food Prot..

[29] Jonasson S (2010). Transfer of a cyanobacterial neurotoxin within a temperate aquatic ecosystem suggests pathways for human exposure. Proc. Natl. Acad. Sci..

[30] Bianchi TS (2000). Cyanobacterial blooms in the Baltic Sea: Natural or human-induced?. Limnol. Oceanogr..

[31] Lage S, Annadotter H, Rasmussen U, Rydberg S (2015). Biotransfer of β-N-methylamino-L-alanine (BMAA) in a eutrophicated freshwater lake. Mar. Drugs.

[32] Salomonsson ML, Fredriksson E, Alfjorden A, Hedeland M, Bondesson U (2015). Seafood sold in Sweden contains BMAA: A study of free and total concentrations with UHPLC–MS/MS and dansyl chloride derivatization. Toxicol. Rep..

[33] Bianchi TS, Rolff C, Widbom B, Elmgren R (2002). Phytoplankton Pigments in Baltic Sea Seston and Sediments: Seasonal Variability, Fluxes, and Transformations. Estuar. Coast. Shelf Sci..

[34] Rumsby, P., Hall, T. & Pitchers, R. *Risk assessment of BMAA*. 41 pp. (2008).

[35] Sellner KG, Olson MM, Olli K (1996). Copepod interactions with toxic and non-toxic cyanobacteria from the Gulf of Finland. Phycologia.

[36] Mazur-Marzec H, Tymińska A, Szafranek J, Pliński M (2007). Accumulation of nodularin in sediments, mussels, and fish from the Gulf of Gdańsk, southern Baltic Sea. Environ. Toxicol..

[37] Cabana G, Rasmussen JB (1994). Modelling food chain structure and contaminant bioaccumulation using stable nitrogen isotopes. Nature.

[38] Borgå K (2012). Trophic magnification factors: Considerations of ecology, ecosystems, and study design. Integr. Environ. Assess. Manag..

[39] Mak YL (2013). Pacific Ciguatoxins in Food Web Components of Coral Reef Systems in the Republic of Kiribati. Environ. Sci. Technol..

[40] Spilling, K. *et al*. Shifting Diatom—Dinoflagellate Dominance During Spring Bloom in the Baltic Sea and its Potential Effects on Biogeochemical Cycling. *Front. Mar. Sci*. **5** (2018).

[41] Wasmund N (1997). Occurrence of cyanobacterial blooms in the Baltic Sea in relation to environmental conditions. Int. Rev. Gesamten Hydrobiol. Hydrogr..

[42] Aarnio K, Bonsdorff E, Rosenback N (1996). Food and feeding habits of juvenile flounder Platichthys flesus (L.), and turbot Scophthalmus maximus L. in the Åland archipelago, northern Baltic Sea. J. Sea Res..

[43] Mustamäki N, Cederberg T, Mattila J (2014). Diet, stable isotopes and morphology of Eurasian perch (Perca fluviatilis) in littoral and pelagic habitats in the northern Baltic Proper. Environ. Biol. Fishes.

[44] Casini M, Cardinale M, Arrhenius F (2004). Feeding preferences of herring (Clupea harengus) and sprat (Sprattus sprattus) in the southern Baltic Sea. ICES J. Mar. Sci..

[45] Sivonen K (1989). Occurrence of the hepatotoxic cyanobacterium Nodularia spumigena in the Baltic Sea and structure of the toxin. Appl. Environ. Microbiol..

[46] Faassen EJ, Gillissen F, Zweers HAJ, Lürling M (2009). Determination of the neurotoxins BMAA (β-N-methylamino-L-alanine) and DAB (α-,γ-diaminobutyric acid) by LC-MSMS in Dutch urban waters with cyanobacterial blooms. Amyotroph. Lateral Scler..

[47] Kiørboe T (2013). Zooplankton body composition. Limnol. Oceanogr..

[48] Sládeček V, Sládečková A (1963). Relationship between wet weight and dry weight of the periphyton. Limnol. Oceanogr..

[49] Metcalf JS (2008). Co-occurrence of beta-N-methylamino-L-alanine, a neurotoxic amino acid with other cyanobacterial toxins in British waterbodies, 1990–2004. Environ. Microbiol..

[50] Esterhuizen M, Downing TG (2008). Beta-N-methylamino-L-alanine (BMAA) in novel South African cyanobacterial isolates. Ecotoxicol. Environ. Saf..

[51] Christensen SJ, Hemscheidt TK, Trapido-Rosenthal H, Laws EA, Bidigare RR (2012). Detection and quantification of β-methylamino-L-alanine in aquatic invertebrates: BMAA analysis in aquatic invertebrates. Limnol. Oceanogr. Methods.

[52] Faassen, E. J. *et al*. A Collaborative Evaluation of LC-MS/MS Based Methods for BMAA Analysis: Soluble Bound BMAA Found to Be an Important Fraction. *Mar. Drugs* **14** (2016). 10.3390/md14030045 PMC4820299 26938542

[53] Masseret E (2013). Dietary BMAA Exposure in an Amyotrophic Lateral Sclerosis Cluster from Southern France. PLOS ONE.

[54] Motwani NH, Gorokhova E (2013). Mesozooplankton Grazing on Picocyanobacteria in the Baltic Sea as Inferred from Molecular Diet Analysis. PLoS ONE.

[55] Kozlowsky-Suzuki B (2003). Feeding, reproduction and toxin accumulation by the copepods Acartia bifilosa and Eurytemora affinis in the presence of the toxic cyanobacterium Nodularia spumigena. Mar. Ecol. Prog. Ser..

[56] Gismervik I (2006). Top-down impact by copepods on ciliate numbers and persistence depends on copepod and ciliate species composition. J. Plankton Res..

[57] Rudstam LG, Danielsson K, Hansson S, Johansson S (1989). Diel vertical migration and feeding patterns of Mysis mixta (Crustacea, Mysidacea) in the Baltic Sea. Mar. Biol..

[58] Walters DM (2016). Trophic Magnification of Organic Chemicals: A Global Synthesis. Environ. Sci. Technol..

