# Supplementary material for: Insufficient evidence for BMAA transfer in the pelagic and benthic food webs in the Baltic Sea
Source: Sci Rep. 2019 Jul 18;9:10406. doi: 10.1038/s41598-019-46815-3 (PMC6639344; doi:10.1038/s41598-019-46815-3)
Supplement: Supplementary file 1 — Supplementary Information [file 41598_2019_46815_MOESM1_ESM.docx]

**Supplementary Information**

**Insufficient evidence for BMAA transfer in the pelagic and benthic food webs in the Baltic Sea**

Nadezda Zguna^a^, Agnes M. L. Karlson^a,b^, Leopold L. Ilag^a^, Andrius Garbaras^c^, Elena Gorokhova^a*^

^a^Department of Environmental Science and Analytical Chemistry, Stockholm University, Sweden

^b^Department of Ecology, Environment and Plant Science, Stockholm University, Sweden

^c^Mass Spectrometry Laboratory, Center for Physical Science and Technology, Vilnius, Lithuania

*Correspondence author: [elena.gorokhova@aces.su.se](mailto:elena.gorokhova@aces.su.se) *

4 pages, 2 figures

**Figure S1.** Map of the Askö area with three sampling stations used for collecting benthic invertebrates and sediment (Uttervik [U] and Hålldämman [H]) and phytoplankton (B1). See Fig. 1 for the map with all stations used in this study. The map was generated by SGU (Geological Survey of Sweden; the copyright holder) using Map generator service: <http://www.sgu.se/en/products/maps/map-generator>; accessed: 2019-03-01;


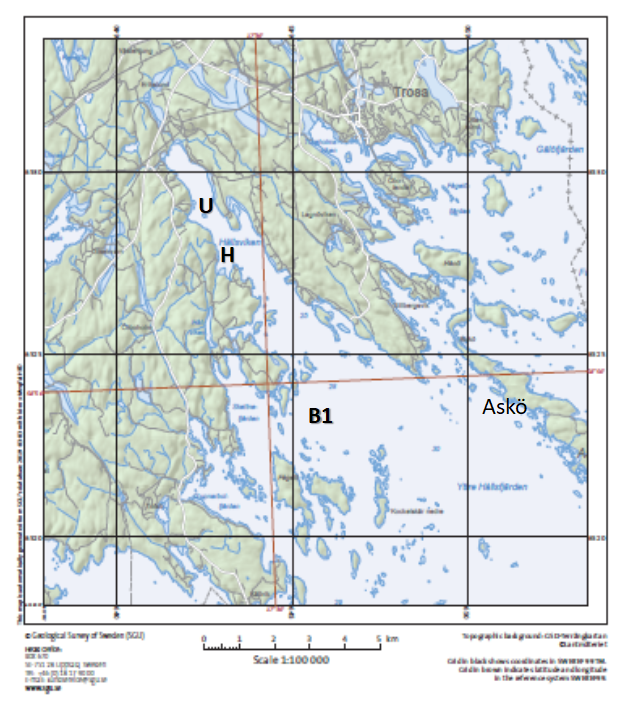


**Figure S2.** LC-MS/MS chromatograms of BMAA and its isomer standards (5 mg L^–1^ for BAMA, BMAA, and AEG and 20 mg L^–1^ for DAB), and chromatograms showing BMAA produced by reference samples (blue mussel and axenic culture of *Nodularia spumigena*), spiked cod (panel A), and test samples (zooplankton and seston; panel B).

(A)

| 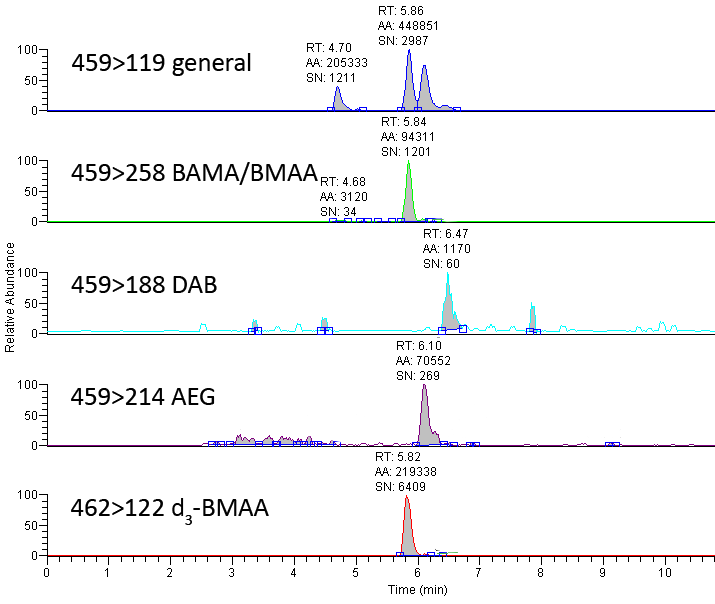 | 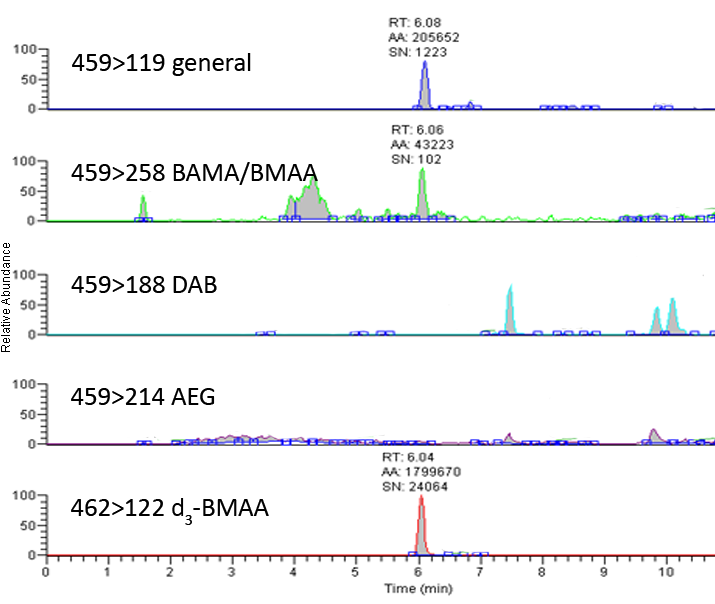 |
| --- | --- |
| standard | blue mussel |
| 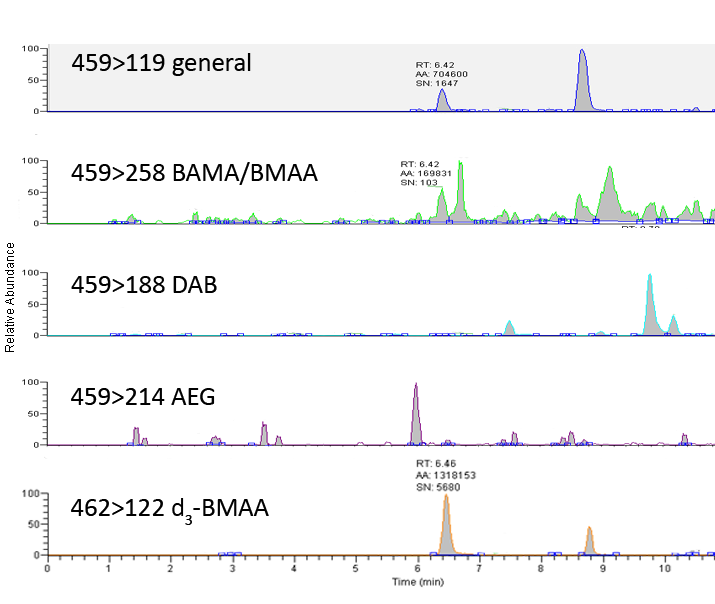 | 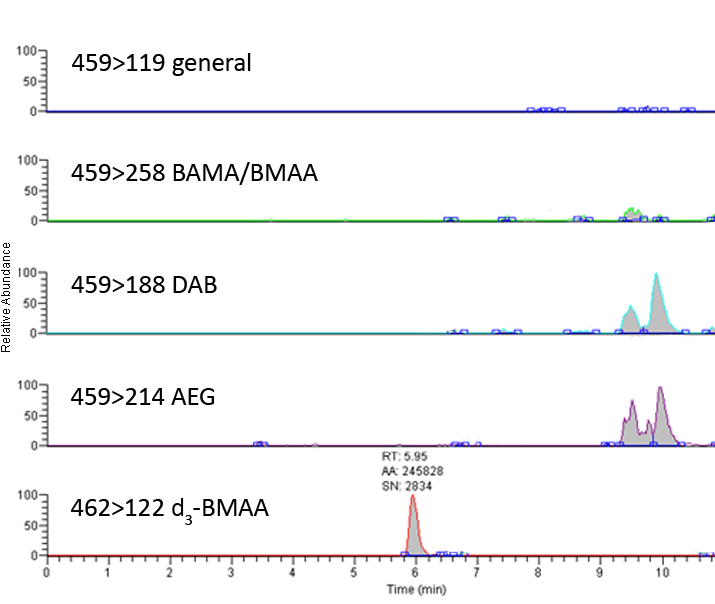 |
| *Nodularia spumigena* | cod |

**Figure S2** Continued.

(B)

| 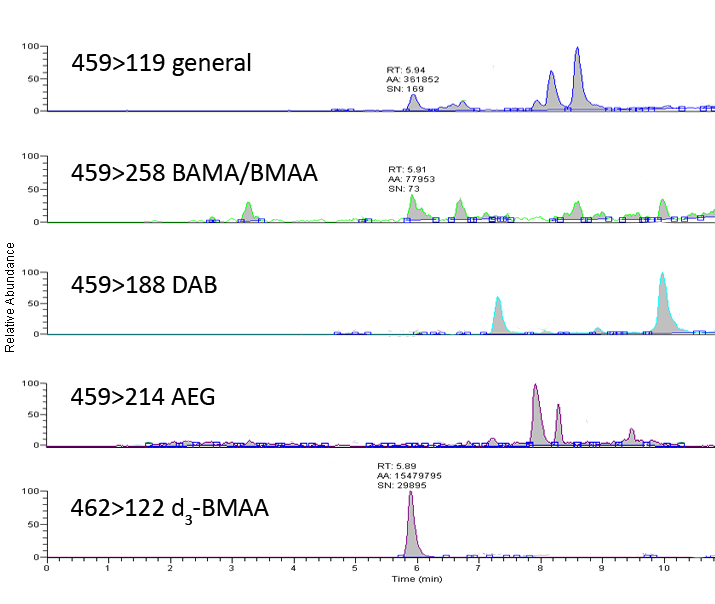 | 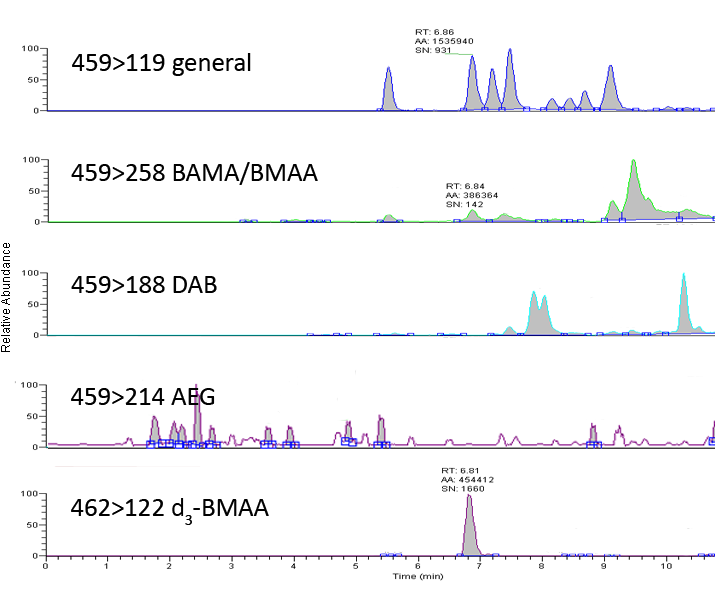 |
| --- | --- |
| zooplankton | seston |
